# The Extraction of Cataract

**Published:** 1907-05-25

**Authors:** 


### Ophthalmology.

### THE EXTRACTION OF CATARACT.

When should a patient be advised to undergo an operation for the removal of a senile cataract?

The answer to this question would be quite simple if patients possessed only one eye, as then the advice would depend upon the degree to which vision was compromised; whereas, with two eyes, the condition of the vision in the other eye affects the expression of an opinion very considerably. A cataract is removed most easily, and with the greatest benefit to the patient, when it is ripe, that is, where there is complete opacity of the lens. At this stage the patient's sight in the affected eye is reduced to the mere perception and projection of light, and the operation will probably be a success, that is to say, the patient will see better after the operation than before. By "perception of light" is meant the ability of the patient to see through his cataract a lighted candle held at a few feet from his face, and by "projection of light" is meant his ability to locate accurately the position of the light in his field of vision. This amount of vision *must* be present. Without it an operation is not advisable, as the fundus of the eye in such circumstances will probably be diseased. Optic atrophy, choroiditis, detachment of the retina, or some other affection may be concealed behind the opaque lens.

If, then, the patient has a mature cataract in one eye, and has perception and projection of light in that eye, should he be advised to undergo an operation? The answer to that question depends entirely upon the visual acuity of the other eye.

If the other eye is blind, or the vision is, from some cause or another, so defective that the patient is unable to read or to move about safely, an extraction should be suggested, but if he sees well with the other eye and can read and get about, there is no necessity for any immediate operation. Should a mature cataract be present in one eye it is not necessary to extract it, provided that six-eighteenths or still better vision exists in the other eye. The reason for this is that the good eye will always be more serviceable than the eye which has been operated on, even though the latter has a greater visual acuity, as the sound eye retains its focussing power, which an eye deprived of its lens has lost. A high convex lens for distant vision and a still higher one for near vision is always required after the extraction of a cataract, and thus the two eyes no longer act in association for all distances.

Still less is an operation indicated where one eye contains a mature cataract and the other remains quite healthy and with normal vision. An operation is only justifiable in this case if the patient wishes it because the unusual appearance of his eye diminishes his chances of employment, or on account of some other economic or social reasons; it cannot be urged for visual reasons. Vision is measured by the visual acuity of the better eye, so that if the visual acuity of one eye is quite good, the removal of a cataract from the other eye may not increase the acuity of vision, but only the area of the visual field. Since successful vision in an eye deprived of its crystalline lens usually depends on the use of a high convex glass, the patient's power of orientation is much diminished by the prismatic effect of this convex glass in lateral vision and by the absence of accommodative power in the eye, although his actual visual acuity may equal six-sixths.

If the cataract is not ripe in either eye the necessity for operation is still less apparent. It may be argued—If the operation will at some time be necessary why not operate at once? The answer is that patients may still retain useful vision for years, and may even die before the necessity for an operation arises. A house need not be demolished on the first sign of a flaw in its structure, or a lethal chamber advised for every one showing signs of old age.

The rapidity with which a lens becomes entirely opaque varies with age and other conditions. Lens opacities increase much more rapidly in young than in old patients, and more rapidly in hypermetropes than in myopes. The conclusions may be tabulated thus:—

| *Present Condition.* | *To Operate or Not?* |
|---|---|
| 1. Mature cataract in an otherwise healthy eye with vision equal to $\frac{6}{6}$ in the other eye. | No operation should be advised except for the sake of appearance. |
| 2. Mature cataract in one eye, and $\frac{6}{18}$ or better in the other. | No operation should be performed. |
| 3. Mature cataract in one eye, and $\frac{6}{24}$ or worse in the other. | Operation may be advised on the mature cataract. |
| 4. Immature cataract in each eye, the visual acuity being equal to $\frac{6}{18}$ or more. | An operation is not necessary, unless the cataract is progressing rapidly. |
| 5. Immature cataract in each eye, the visual acuity being less than $\frac{6}{18}$. | An operation should be performed on the eye with the poorer vision. |

In conclusion, there is no urgency for an operation in any case when the patient's visual acuity is six-eighteenths, or can be brought up to six-eighteenths with suitable glasses. Patients having this vision can usually read ordinary print. If the cataract is increasing rapidly there is more reason for operative interference than if it is slow.

Never omit to examine a case of suspected cataract as early as possible, and that for two reasons: First, the diagnosis may not be correct, and cases of glaucoma have been watched until vision was irrevocably lost under the impression that they were cases of cataract; and secondly, the surgeon ought always to take an opportunity of examining the fundus in order to see if it is healthy before the lens becomes too opaque for this to be done.

